# Reallocating time between device-measured 24-hour activities and cardiovascular risk in Asian American immigrant women: An isotemporal substitution model

**DOI:** 10.1371/journal.pone.0297042

**Published:** 2024-01-10

**Authors:** Chorong Park, Britta Larsen, Simona C. Kwon, Yuhe Xia, Marianna LaNoue, Victoria V. Dickson, Harmony R. Reynolds, Tanya M. Spruill

**Affiliations:** 1 School of Nursing, Vanderbilt University, Nashville, Tennessee, United States of America; 2 Sarah Ross Soter Center for Women’s Cardiovascular Research, New York University Grossman School of Medicine, New York, New York, United States of America; 3 Department of Family Medicine and Public Health, School of Medicine, University of California, San Diego, La Jolla, California, United States of America; 4 Department of Population Health, New York University Grossman School of Medicine, New York, New York, United States of America; 5 New York University Rory Meyers School of Nursing, New York, New York, United States of America; 6 School of Nursing, University of Connecticut, Storrs, Connecticut, United States of America; Tehran University of Medical Sciences, ISLAMIC REPUBLIC OF IRAN

## Abstract

The 24-hour day consists of physical activity (PA), sedentary behavior, and sleep, and changing the time spent on one activity affects the others. Little is known about the impact of such changes on cardiovascular risk, particularly in Asian American immigrant (AAI) women, who not only have a higher cardiovascular risk but also place greater cultural value on family and domestic responsibilities compared to other racial/ethnic groups. The purpose of this study was to evaluate the effects of reallocating 30 minutes of each 24-hour activity component for another on BMI, waist circumference, and blood pressure in AAI women. Seventy-five AAI women completed 7 days of hip and wrist actigraphy monitoring and were included in the analysis (age = 61.5±8.0 years, BMI = 25.5±3.6 kg/m^2^, waist circumference = 85.9±10.2 cm). Sleep was identified from wrist actigraphy data, and moderate-to-vigorous PA (MVPA), light PA, and sedentary behavior identified from hip actigraphy data. On average, the women spent 0.5 hours in MVPA, 6.2 hours in light PA, 10 hours in sedentary activities, and 5.3 hours sleeping within a 24-hour day. According to the isotemporal substitution models, replacing 30 minutes of sedentary behavior with MVPA reduced BMI by 1.4 kg/m^2^ and waist circumference by 4.0 cm. Replacing that same sedentary time with sleep reduced BMI by 0.5 kg/m^2^ and waist circumference by 1.4 cm. Replacing 30 minutes of light PA with MVPA decreased BMI by 1.6 kg/m^2^ and waist circumference by 4.3 cm. Replacing 30 minutes of light PA with sleep also reduced BMI by 0.8 kg/m^2^ and waist circumference by 1.7 cm. However, none of the behavioral substitutions affected blood pressure. Considering AAI women’s short sleep duration, replacing their sedentary time with sleep might be a feasible strategy to reduce their BMI and waist circumference.

## Introduction

Cardiovascular disease (CVD) is one of the most prevalent diseases (45%) among Asian American women and a major cause of morbidity and mortality [1]. Although Asian American immigrant (AAI) women generally have a lower BMI than non-Hispanic Whites, they face elevated CVD risks, including central obesity, high adiposity, elevated lipid levels, and an increased risk of diabetes [2–5]. Asian Americans, especially in women, have higher prevalence of low HDL-C and high triglycerides, compared to non-Hispanic Whites [6]. A meta-analysis highlighted that Asians without diabetes have higher HbA1c compared to non-Hispanic Whites by a difference of 0.24% (2.6 mmol/mol). This trend is further underscored by the higher diabetes prevalence in non-Hispanic Asians (19.1%) relative to non-Hispanic Whites (12.1%), with particular Asian subgroups manifesting even greater prevalence [7]. Additionally, East/Southeast Asians have almost three times the odds of hypertension compared to non-Hispanic White adults [8].

One potential modifiable factor contributing to AAI women’s CVD risk is their unique patterns of 24-hour activity, consisting of light and moderate-to-vigorous levels of physical activity (PA), sedentary behavior, and sleep. Previously, these four behaviors were considered independent cardiovascular risk factors [9, 10], but it is now recognized that they are co-dependent, with changes to one behavior within the 24-hour period resulting in changes to other behaviors [11]. In Asian culture, women are traditionally expected to prioritize family and domestic responsibilities [12–14]. Engaging in PA is often perceived as neglecting these responsibilities [12]. Sleep has been traditionally less prioritized than other behaviors, often sacrificed for other wake-time tasks [15]. Consequently, AAI women spend a significant amount of time on household and caregiving responsibilities, which predominantly involve light intensity PA [13, 16]. They also tend not to engage in leisure time PA [14] and typically have shorter sleep durations [15, 17]. Indeed, with two accelerometers, we previously reported that AAI women have 10 hours/day of sedentary time and 5.3 hours/day of sleep duration, which is less than the averages of the general U.S. population [18].

Given the distinct 24^-^hour activity patterns observed in AAI women, the effects of replacing one activity with another on CVD risk factors might differ compared to other racial/ethnic groups. For example, in studies using UK Biobank or US NHANES 2005–2006 data, which include predominantly White men and women, reallocating 30 minutes of sedentary behavior or sleep with MVPA was associated with more favorable adiposity measures [19, 20]. However, these findings might not be applicable to AAI women who have very short sleep duration. An increase in MVPA must result in a decrease in another behavior, and a large portion of other wake time activities in AAI women is related to gender roles such as household and family care [16]. Therefore, advising AAI women to increase MVPA might not have beneficial effects if they do this by reducing sleep time, rather than by reducing sedentary time. To optimize their 24-hour activity patterns and develop tailored interventions, it is crucial to understand time reallocation effects on cardiovascular risk in diverse AAI women. Therefore, the aim of this study was to evaluate the effects of reallocating 30 minutes of each 24-hour activity component for another on cardiovascular risk factors (BMI, waist circumference, and blood pressure) in AAI women.

## Methods

We recruited Asian American immigrant women currently living in New York City (NYC). We focused on the three largest Asian subgroups in NYC: East Asians (i.e., Chinese and Koreans), Southeast Asians (i.e., Filipinos), and South Asians (i.e., Bangladeshis) [21]. The eligibility criteria were (1) age between 18 and 75; (2) self-identified as an AAI woman born in one of four countries (i.e., China, Bangladesh, Philippines, South Korea) and immigrated to the U.S.; (3) not currently being treated with antihypertensive medications; (4) ability to read, speak, and write in English, Bangla, Chinese, or Korean; and (5) not currently pregnant.

We calculated the minimum sample size needed to detect the effect of replacing 30 minutes of sedentary time with MVPA on BMI. We used the effect estimate from a meta-analysis [22] which found that replacing 30 minutes of sedentary time with MVPA had a significant effect on BMI, with a pooled effect size of beta = –1.07 (95% CI = –1.80, –0.3). Based on this information, a minimum sample size of 80 was required to detect this effect within 95% CI with a power of > 0.8 to show the estimated required sample size for detecting these effects.

Between August 2018 and August 2019, participants were recruited from Asian faith-based organizations and community-based organizations located in New York City. The detailed recruitment procedure is described elsewhere [18] and was approved by the institutional review board of NYU Grossman School of Medicine (IRB # i18-00268). We adhered to the principles of the Helsinki Declaration, obtained informed consent from participants after explaining the study’s purpose, potential risks, and benefits, and ensured the confidentiality and privacy of all participants throughout the study. The informed consent and all study materials including surveys and accelerometer wearing instructions were developed in English, Korean, Chinese, and Bangla and explained by trained bilingual research assistants.

In order to objectively measure the entire intensity spectrum of 24-hour activity, we used both wrist-accelerometry (a criterion measure of sleep in free-living settings) [23] and hip-accelerometry (a criterion measure of PA and sedentary behavior in free-living settings) [24]. For 7 consecutive days, participants wore two triaxial accelerometers (ActiGraph GT3X; ActiGraph Corp., Pensacola, Florida) for the entire 24-hour period. Accelerometer data processing was performed using the ActiLife software (version 6.13.3, ActiGraph Corp.) as described elsewhere [18]. In summary, the sleep period was identified from wrist accelerometer data, using the Cole-Kripke algorithm [25] and a sleep diary. From these sleep periods, the minutes of “asleep” were summed to calculate sleep duration. Next, after excluding the sleep period from the hip accelerometer data, we applied the Choi et al. algorithm to identify non-wear time [26]. Freedson’s cutoff points were used to classify time spent in MVPA (≥1952 counts/min), light PA (101–1951 counts/min), and sedentary behavior (≤100 counts/min) [27] based on the valid wear time data from the hip accelerometer. For the analysis, participants who had a minimum of 4 days with 10 hours/day of wear time of both accelerometers from midnight-to-midnight were included in the current analyses.

Outcome variables included BMI, waist circumference, and blood pressure. Height and weight were measured without shoes or heavy clothing and BMI was calculated by weight(kg)/height(m)^2^. Overweight and obesity were defined by using Asian-specific BMI cutoffs (overweight: BMI ≥ 23, obesity: BMI ≥ 27.5) [28]. Waist circumference was measured horizontally around the waist 2 cm above the umbilicus. Central obesity was defined by using Asian women’s waist circumference ≥ 80cm [29]. Blood pressure was measured using an automated and validated blood pressure monitor (Omron HEM-705CP) [30]. Using American Heart Association blood pressure measurement recommendations, 3 blood pressure measurements were obtained at 2-minute intervals and the average of the 3 measurements was used in the analysis [31].

Covariates included age, education level (college or higher), and marital/partner status (yes/no) and comorbidity. The Comorbidity Questionnaire [32] was used to assess 12 medical conditions including heart disease, high blood pressure, diabetes, cancer, and depression. A higher total score indicates greater comorbidity. This questionnaire has acceptable convergent validity with the chart-based Charlson Comorbidity Index (Spearman’s rho = 0.55) and excellent test-retest reliability (Intraclass Correlation Coefficient [ICC] = 0.94) [32].

For analysis, all 24-hour activity variables were converted to 30 minutes/day units to aid in interpretation of the regression coefficients. All outcome variables were examined for normality and these variables approximated a normal distribution. We conducted a three-step analysis to test the reallocation effects on CVD risk factors. First, a single regression model examined the raw associations of each intensity category (sleep, sedentary activity, light PA, and MVPA) with each CVD risk factor without mutual adjustment for other categories of activity. Second, a partition model examined the associations of each intensity category with each CVD risk factor after adjusting for all other 24-hour activity variables. Finally, an isotemporal substitution model was used to evaluate the reallocation effects of one 24-hour activity component with other activity components [33]. The isotemporal substitution model estimates the effects of reallocating one 24-hour activity component for another (e.g., 30 minutes of sedentary behavior to MVPA) on each CVD risk factor, while simultaneously adjusting for the confounding effect of the remaining 24-hour activity components [34]. This was accomplished by including a total wear time variable in the model and dropping the exposure variable of interest (e.g. sedentary behavior). The coefficient can be interpreted as the effect of replacing 30 minutes of sedentary behavior with the respective other activity in the regression (e.g., 30 minutes of MVPA). For example, BMI = (β0) Light PA + (β1) Moderate-to-vigorous PA + (β2) sleep + (β3) total wear time + (β4) covariates. The coefficient (β0) shows the change in BMI when sedentary behavior is replaced with 30 minutes of light PA per day. Similarly, the coefficient (β1) depicts the BMI change when 30 minutes of sedentary behavior is replaced with 30 minutes of moderate-to-vigorous PA daily. The same interpretive approach can be used for other substitution models, accounting for any activity component that is excluded from the equation. Results are presented as unstandardized regression coefficients. Multicollinearity among variables in all models was tested by using variance inflation factors (VIF <2.5). The statistical level was set at *p* < 0.05 and was two-sided. All analyses were cross-sectional, and performed using IBM SPSS 28.0 (SPSS Inc., Chicago, IL, USA) and R (version 4.2.0, https://www.r-project.org/).

We encountered 13% of missing self-report data (n = 11, 13%) which included demographic and clinical characteristics (age, education levels, marital status, and comorbidity index). The missing data were not missing at random; the majority of the missing data was from one Asian subgroup (Bangladeshi) due to their low levels of written Bengali literacy. Because the number of missing values for these variables would result in an unacceptable loss of cases in modeling, these variables were not included in modeling. In supplementary analyses, we re-ran all models while adjusting for these demographic variables in the reduced sample.

## Results

Eighty-six participants were eligible for the study of which 75 (87%) completed both hip and wrist accelerometer monitoring for at least 4 days and were included in the analysis. Participants’ total hip-accelerometer wear time was 1272.9 (SD = 233.6) minutes/day and wrist-accelerometer wear time was 1347.1 (SD = 216.1) minutes/day. Demographics and CVD risk factors are shown in Table 1. Average minutes per day spent in each category of 24-hour behaviors are also shown in Table 1.

**Table 1:** Characteristics of demographics and comorbidity, cardiovascular disease risk factors, and 24-hour activity components (N = 75).

Variables	Mean ± SD or %
**Demographics and Clinical Characteristics**	
Age (years)	61.36 ± 7.92
Asian subgroup (%)	
East Asians	51%
Southeast Asians	24%
South Asians	25%
College graduate (%)	57%
Employed (%)	49%
Married (%)	80%
Comorbidity Index (Median, [IQR])	2 [0, 3]
**Cardiovascular Disease Risk Factors**	
BMI (kg/m^2^)	25.52 ± 3.62
% Overweight or obese*	76%
Waist (cm)	85.94 ± 10.17
% Central obesity†	71%
Systolic blood pressure (mm Hg)	130.72 ± 14.91
Diastolic blood pressure (mm Hg)	76.81 ± 10.10
% Stage 1 Hypertension‡	31%
% Stage 2 Hypertension‡	35%
**24-hour Activity Components**	
Daily Sedentary (minutes/day)	595.00 ± 108.97
Daily Light Activity (minutes/day)	369.46 ± 101.33
Daily MVPA (minutes/day)	32.89 ± 21.92
Daily Sleep Duration (minutes/day)	316.93 ± 50.42
Daily Time Spent in Bed (minutes/day)	406.79 ± 56.09

Abbreviation: BMI = Body mass index, IQR = interquartile range

* Overweight: BMI ≥ 23, Obesity: BMI ≥ 27.5 [28]

†Central obesity: Asian women’s waist circumferences ≥ 80cm [29]

‡Stage 1 Hypertension: 130 to 139 mm Hg systolic or 80 to 89 mm Hg diastolic, Stage 2 Hypertension: at least 140 mm Hg systolic or at least 90 mm Hg diastolic [31]

More than half of participants (65.3%) met the 2018 PA guideline of at least 150 minutes of moderate PA or 75 minutes of vigorous PA per week [35]. Average sleep duration and sleep efficiency were below recommendations; only 1.3% of women met the recommended sleep duration (≥ 7 hours duration [36]) and 81.3% of women had poor sleep efficiency (< 85% efficiency [37]). There were low-to-moderate correlations between the different activity categories; the lowest correlation was between sleep duration and MVPA time (r = 0.07, *p* = 0.55) and the highest correlation was between light PA and sedentary time (r = 0.69, *p* < 0.01). There was no indication of multicollinearity among variables in all models; all variance inflation factors were < 2.5.

### Activity substitution effects on BMI

Table 2 presents coefficients for the BMI models. In single models, greater time in MVPA and sleep were each associated with lower BMI, whereas sedentary behavior and light PA were not significantly associated with BMI. In the partition models, where the time in each of the intensity categories was held constant, MVPA and sleep remained negatively associated with BMI. Lastly, in isotemporal substitution models that held total time constant, replacing 30 minutes of sedentary time with MVPA was associated with a -1.37kg/m^2^ (95% CI: -2.48, -0.26) decrease in BMI. Replacing 30 minutes of sedentary time with sleep was also associated with a -0.52 kg/m^2^ (95% CI: -0.99, -0.05) decrease in BMI, whereas replacing 30 minutes of sedentary time with light PA was not significantly associated with BMI. In addition, replacing 30 minutes of light PA with MVPA or sleep was significantly associated with a decrease in BMI. Replacing 30 minutes of sleep with MVPA was not significantly associated with BMI.

**Table 2:** Single, partition, and isotemporal substitution models examining the relation between 30-min changes in 24-hour activity components and BMI (N = 75).

Model	Sedentary	Light PA	MVPA	Sleep
**Single**	0.02 (-0.21, 0.25)	0.12 (-0.13, 0.36)	-1.24 (-2.35, -0.13)**	-0.63 (-1.11, -0.15)**
**Partition**	-0.24 (-0.64, 0.16)	-0.01 (-0.39, 0.37)	-1.61 (-2.80, -0.43)**	-0.76 (-1.35, -0.18)*
**Isotemporal substitution**				
Replace sedentary	NA	0.23 (-0.03, 0.49)	-1.37 (-2.48, -0.26)*	-0.52 (-0.99, -0.05)*
Replace light PA	-0.23 (-0.49, 0.03)	NA	-1.6 (-2.81, -0.4)**	-0.75 (-1.25, -0.25)**
Replace MVPA	1.37 (0.26, 2.48)*	1.6 (0.4, 2.81)**	NA	0.85 (-0.40, 2.10)
Replace sleep	0.52 (0.05, 0.99)*	0.75 (0.25, 1.25)**	-0.85 (-2.10, 0.40)	NA

*Note*. Values represent unstandardized regression coefficients (95% Confidence Intervals).

Abbreviation: NA = not applicable, PA = physical activity, MVPA = Moderate-to-vigorous physical activity

*p < 0.05

**p < 0.01.

### Activity substitution effects on waist circumference

Table 3 presents coefficients for the various waist circumference models. Greater time in MVPA and sleep were associated with smaller waist circumference in the single and partition models. In isotemporal substitution models, replacing 30 minutes of sedentary time with MVPA was associated with a -3.96 cm (95% CI: -7.15, -0.76) decrease in waist circumference. Replacing 30 minutes of sedentary time with sleep was also associated with a -1.4 cm (95% CI: -2.76, -0.04) decrease in waist circumference. Replacing 30 minutes of light PA with MVPA or sleep was also significantly associated with a decrease in waist circumference. Replacing 30 minutes of sleep with MVPA was not significantly associated with waist circumference.

**Table 3:** Single, partition, and isotemporal substitution models examining the relation between 30-min changes in 24-hour activity components and waist circumference (N = 75).

Model	Sedentary	Light PA	MVPA	Sleep
**Single**	0.37 (-0.28, 1.02)	0.21 (-0.50, 0.91)	-3.77 (-6.90, -0.65)*	-1.4 (-2.76, -0.04)*
**Partition**	0.14 (-1.00, 1.29)	0.45 (-0.66, 1.55)	-3.82 (-7.22, -0.41)*	-1.26 (-2.95, 0.43)
**Isotemporal substitution**				
Replace sedentary	NA	0.3 (-0.45, 1.06)	-3.96 (-7.15, -0.76)*	-1.4 (-2.76, -0.04)*
Replace light PA	-0.3 (-1.06, 0.45)	NA	-4.26 (-7.72, -0.8)*	-1.7 (-3.14, -0.27)*
Replace MVPA	3.96 (0.76, 7.15)*	4.26 (0.8, 7.72)*	NA	2.56 (-1.04, 6.16)
Replace sleep	1.4 (0.04, 2.76)*	1.7 (0.27, 3.14)*	-2.56 (-6.16, 1.04)	NA

*Note*. Values represent unstandardized regression coefficients (95% Confidence Intervals).

Abbreviation: NA = not applicable, PA = physical activity, MVPA = Moderate-to-vigorous physical activity

*p < 0.05

**p < 0.01.

### Activity substitution effects on blood pressure

None of the activity categories were associated with systolic or diastolic blood pressure in any of the single, partition or isotemporal substitution models. The detailed results are included in Table 4.

**Table 4:** Single, partition, and isotemporal substitution models examining the relation between 30-min changes in 24-hour activity components and systolic and diastolic blood pressure (N = 75).

Model	Sedentary	Light PA	MVPA	Sleep
**Systolic Blood Pressure**				
**Single**	0.23 (-0.73, 1.18)	-0.16 (-1.19, 0.87)	-1.03 (-5.79, 3.72)	-0.82 (-2.88, 1.24)
**Partition**	-0.27 (-2.10, 1.53)	-0.38 (-2.11, 1.36)	-0.96 (-6.33, 4.40)	-1.08 (-3.74, 1.58)
**Isotemporal substitution**				
Replace sedentary	NA	-0.11 (-1.3, 1.08)	-0.69 (-5.72, 4.34)	-0.81 (-2.95, 1.32)
Replace light PA	0.11 (-1.08, 1.3)	NA	-0.58 (-6.04, 4.87)	-0.7 (-2.96, 1.55)
Replace MVPA	0.69 (-4.34, 5.72)	0.58 (-4.87, 6.04)	NA	-0.12 (-5.79, 5.55)
Replace sleep	0.81 (-1.32, 2.95)	0.7 (-1.55, 2.96)	0.12 (-5.55, 5.79)	NA
**Diastolic Blood Pressure**				
**Single**	-0.24 (-0.89, 0.40)	0.28 (-0.42, 0.97)	-0.78 (-4.00, 2.44)	-0.54 (-1.94, 0.85)
**Partition**	-0.69 (-1.89, 0.52)	-0.20 (-1.36, 0.96)	-1.79 (-5.38, 1.80)	-1.04 (-2.82, 0.74)
**Isotemporal substitution**				
Replace sedentary	NA	0.48 (-0.31, 1.28)	-1.11 (-4.47, 2.26)	-0.35 (-1.78, 1.08)
Replace light PA	-0.48 (-1.28, 0.31)	NA	-1.59 (-5.24, 2.06)	-0.84 (-2.35, 0.67)
Replace MVPA	1.11 (-2.26, 4.47)	1.59 (-2.06, 5.24)	NA	0.75 (-3.04, 4.55)
Replace sleep	0.35 (-1.08, 1.78)	0.84 (-0.67, 2.35)	-0.75 (-4.55, 3.04)	NA

*Note*. Values represent unstandardized regression coefficients (95% Confidence Intervals).

Abbreviation: NA = not applicable, PA = physical activity, MVPA = Moderate-to-vigorous physical activity

*p < 0.05

**p < 0.01.

### Supplementary analysis: Adjusted model with demographic covariates

The adjusted model indicated that results remained consistent when we adjusted the BMI, waist circumference, and blood pressure models for age, education level, marital status, and comorbidity. The detailed results are included in S1 Table.

## Discussion

To our knowledge, this is the first study showing the effects of reallocating 24-hour activity behaviors on CVD risk factors in diverse subgroups of AAI women. In this study, isotemporal substitution models demonstrated a clinically relevant effect of replacing sedentary behavior or light PA with either sleep or MVPA in AAI women. Considering that our sample of AAI women spent an average of 30 minutes in MVPA per day but only spent 5.3 hours in sleep, replacing 30 minutes of sedentary time with sleep might be a more feasible strategy to improve cardiovascular risk than encouraging them to engage in more MVPA.

The degree of change in BMI or waist circumference obtained in our model is modest but would have a measurable effect on cardiovascular risk. We acknowledge that our initial target was to recruit 80 participants to detect a -1.07 effect on BMI from reallocating 30 minutes of sedentary time to moderate-to-vigorous PA. We analyzed data from 75 participants, excluding those who did not wear hip and wrist actigraphy for at least 4 days, following our wear-time protocol. However, our observed effect was a more significant -1.69 (95% C.I.: -2.86, -0.53), which was still detectable with our sample size (*p* = .005).

Modeling results indicated that substituting 30 minutes of sedentary behavior or light PA with 30 minutes of MVPA or sleep could significantly reduce AAI women’s BMI by a median of 0.5–1.6 kg/m^2^ and waist circumference by 1.4–4.3 cm (= 0.6–1.7 inches). A reduction of 1.6 kg/m^2^ in BMI is equivalent to a 6% reduction in body weight based on the sample mean; a weight reduction of 5% has been confirmed to reduce the risk of future CVD events [38, 39], highlighting the potential benefit of relatively small behavior changes. A reduction of 4.3 cm in waist circumference can also potentially lower cardiovascular risk, given a 1 cm increase in waist circumference was associated with a 2% increase in risk of a future CVD event [40, 41]. These findings set the stage for development of tailored CVD prevention interventions for AAI women, a group known to be at increased risk of CVD.

It is well known that reallocating sedentary time to MVPA results in the largest reduction in BMI and waist circumference among all possible behavioral reallocations [22]. However, there are mixed findings on the effects of reallocating sedentary time with sleep: several studies that used objective measures of sleep have shown significant reallocation effects of sedentary behavior with sleep in adiposity parameters [42, 43], but most studies have not shown a significant adiposity reduction due to use of only self-reported sleep duration or even omitted sleep duration in their isotemopral substitution modeling [33]. In our study, we concurrently used wrist and hip accelerometers, valid measures of sleep and PA, and found 1) significant BMI and waist circumference reduction when reallocating time from sedentary behavior to sleep and 2) no significant BMI and waist circumference reduction when reallocating time from sleep to MVPA or vice versa. These two findings suggest that reallocating 30 minutes from sedentary time to sleep or MVPA may have similar beneficial effects on BMI and waist circumference in AAI women who have insufficient sleep duration (<7 hours per day). This finding is aligned with findings from the Women’s Health Initiative Long Life Study where the significant effects of reallocating time from sleep to MVPA were not found in short sleepers [43].

In our study, there were no beneficial effects of replacing sedentary time with light activity, nor was there any evidence to suggest effects of light activity on CVD risk factors in single models. According to a meta-analysis of isotemporal substitution modeling studies, there were beneficial effects of reallocating sedentary behavior with light activity on waist circumference, but not on BMI [22]. These discrepancies might be partly explained by the fact that our sample contained healthy participants with low risk factors and who were already doing a large amount of light PA, mostly related to house chores.

In addition, we did not find significant time reallocation effects on blood pressure. In previous studies, reallocating sedentary time with MVPA was inconsistently related to blood pressure [33]; a study using the 2005–2006 NHANES data also did not find significant time reallocation effects on blood pressure [20], but the Swedish Cardio Pulmonary BioImage Study demonstrated significant effects of reallocating sedentary time with MVPA on blood pressure (OR = 0.92, 95% CI: 0.85, 0.99) [44]. Considering that we excluded those who were taking blood pressure medication, future research with a wider range of blood pressure and a larger population of AAI women is necessary to confirm our findings.

This study has some limitations and interpretation of the findings must be made with caution. First, this is a cross-sectional study and, while the isotemporal models simulate changes, our findings do not imply causal relationships between activity substitutions and CVD risk factors. Additionally, interpretation of the isotemporal substitution model results using a 30-minute unit for each activity does not account for contextual factors such as whether the activity occurred in the morning or evening, or if the 30-minute duration represented a continuous bout or an accumulation of several shorter bouts that together totaled 30 minutes. Future studies should investigate whether the reallocation effects on the outcome variables differ based on the activity’s continuity and timing. Second, our findings should be interpretated with caution due to substantial missing self-administered survey data from the Bangladeshi subgroup. The issue of incomplete Bengali surveys was addressed by omitting significantly impacted demographic characteristics from isotemporal substitution models and conducting sensitivity analyses afterward. We also compared survey-returner vs. non-returner characteristics and found no significant difference at baseline in each of the 24-hour activity components. Third, due to the small sample sizes, we were not able to test the potential differences in effects of behavior substitutions in Asian subgroups. Fourth, although we adjusted our analyses for several key confounders in supplementary analysis, we did not include some other factors that might influence CVD risk or activity patterns, such as dietary intake and occupational status, and cannot rule out the presence of residual confounding. We also did not measure other CVD risk outcomes like fasting glucose, lipids, or HbA1c. Future studies should incorporate these risk outcomes and factors. Lastly, although we proportionally recruited the three largest Asian subgroups in NYC, our findings from our studies have a limited generalizability given that women who participate in the community-based organizations we recruited from may have different physical activity routines than those who do not participate in such organizations.

Despite the limitations, our study findings have important strengths and clinical implications and strengths. We recruited diverse subgroups of AAI women, an underserved and understudied population. We also concurrently used wrist- and hip-accelerometry to objectively measure all the 24-hour components including sleep and light-moderate intensity PA. Our findings on potential BMI and waist circumference benefits of a 30-minute increase in sleep suggest that clinicians and public health providers should consider encouraging AAI women to replace sedentary time with sleep. This may be a more effective strategy than increasing MVPA for AAI women who are highly sedentary and have insufficient sleep, but spend 30 minutes in MVPA daily. Clinical trials are needed to confirm these reallocation effects.

## Conclusions

In this cross-sectional study, we demonstrated that reallocating 30 minutes of sedentary behavior or light PA with MVPA or sleep could significantly reduce AAI womenʼs BMI and waist circumference. We found that a 30-minute increase in sleep had smaller but significant benefits on BMI and waist circumference, compared to an increase in MVPA. Given the short sleep duration in this group, integrating sleep duration and quality assessments into clinical practice is essential. Furthermore, providing a targeted behavior change strategy can contribute to developing clinical guidelines and health policies tailored for AAI women. Results of this study suggest that advising AAI women to replace 30 minutes of sedentary behavior with an equivalent amount of sleep may optimize CVD risk outcomes. Future studies are needed to develop culturally tailored behavioral interventions that promote reallocating 30 minutes of sedentary behavior to sleep and MVPA, and test their effects on CVD risk in Asian American immigrant women.

## Supporting information

S1 Checklist STROBE statement—checklist of items that should be included in reports of observational studies. (DOCX)

S1 Table Supplementary analyses of isotemporal substitution models for cardiovascular risk factors, adjusting for demographic and clinical characteristics (n = 63). (DOCX)
