# Supplementary material for: Reallocating time between device-measured 24-hour activities and cardiovascular risk in Asian American immigrant women: An isotemporal substitution model
Source: PLoS One. 2024 Jan 10;19(1):e0297042. doi: 10.1371/journal.pone.0297042 (PMC10781047; doi:10.1371/journal.pone.0297042)
Supplement: S1 Checklist — (DOCX) [file pone.0297042.s001.docx]

STROBE Statement—checklist of items that should be included in reports of observational studies

|  | Item No. | Recommendation | Page  No. | Relevant text from manuscript |
| --- | --- | --- | --- | --- |
| **Title and abstract** | 1 | (*a*) Indicate the study’s design with a commonly used term in the title or the abstract | 2 | The purpose of this study was to evaluate the effects of reallocating 30 minutes of each 24-hour activity component for another on BMI, waist circumference, and blood pressure in AAI women. |
|  |  | (*b*) Provide in the abstract an informative and balanced summary of what was done and what was found | 2 | See abstract |
| Introduction | | | |  |
| Background/rationale | 2 | Explain the scientific background and rationale for the investigation being reported | 3-4 | Cardiovascular disease (CVD) is one of the most prevalent diseases (45%) among Asian American women and a major cause of morbidity and mortality [1]. Although Asian American immigrant (AAI) women generally have a lower BMI than non-Hispanic Whites, they face elevated CVD risks, including central obesity, high adiposity, elevated lipid levels, and an increased risk of diabetes [2-5]. Asian Americans, especially in women, have higher prevalence of low HDL-C and high triglycerides, compared to non-Hispanic Whites [6]. A meta-analysis highlighted that Asians without diabetes have higher HbA1c compared to non-Hispanic Whites by a difference of 0.24% (2.6 mmol/mol). This trend is further underscored by the higher diabetes prevalence in non-Hispanic Asians (19.1%) relative to non-Hispanic Whites (12.1%), with particular Asian subgroups manifesting even greater prevalence [7]. Additionally, East/Southeast Asians have almost three times the odds of hypertension compared to non-Hispanic White adults [8].  One potential modifiable factor contributing to AAI women’s CVD risk is their unique patterns of 24-hour activity, consisting of light and moderate-to-vigorous levels of physical activity (PA), sedentary behavior, and sleep. Previously, these four behaviors were considered independent cardiovascular risk factors [9, 10], but it is now recognized that they are co-dependent, with changes to one behavior within the 24-hour period resulting in changes to other behaviors [11]. In Asian culture, women are traditionally expected to prioritize family and domestic responsibilities [12-14]. Engaging in PA is often perceived as neglecting these responsibilities [12]. Sleep has been traditionally less prioritized than other behaviors, often sacrificed for other wake-time tasks [15]. Consequently, AAI women spend a significant amount of time on household and caregiving responsibilities, which predominantly involve light intensity PA [13, 16]. They also tend not to engage in leisure time PA [14] and typically have shorter sleep durations [15, 17]. Indeed, with two accelerometers, we previously reported that AAI women have 10 hours/day of sedentary time and 5.3 hours/day of sleep duration, which is less than the averages of the general U.S. population [18].  Given the distinct 24-hour activity patterns observed in AAI women, the effects of replacing one activity with another on CVD risk factors might differ compared to other racial/ethnic groups. For example, in studies using UK Biobank or US NHANES 2005–2006 data, which include predominantly White men and women, reallocating 30 minutes of sedentary behavior or sleep with MVPA was associated with more favorable adiposity measures [19, 20]. However, these findings might not be applicable to AAI women who have very short sleep duration. An increase in MVPA must result in a decrease in another behavior, and a large portion of other wake time activities in AAI women is related to gender roles such as household and family care [16]. Therefore, advising AAI women to increase MVPA might not have beneficial effects if they do this by reducing sleep time, rather than by reducing sedentary time. To optimize their 24-hour activity patterns and develop tailored interventions, it is crucial to understand time reallocation effects on cardiovascular risk in diverse AAI women. Therefore, the aim of this study was to evaluate the effects of reallocating 30 minutes of each 24-hour activity component for another on cardiovascular risk factors (BMI, waist circumference, and blood pressure) in AAI women. |
| Objectives | 3 | State specific objectives, including any prespecified hypotheses | 4 | Therefore, the aim of this study was to evaluate the effects of reallocating 30 minutes of each 24-hour activity component for another on cardiovascular risk factors (BMI, waist circumference, and blood pressure) in AAI women. |
| Methods | | | |  |
| Study design | 4 | Present key elements of study design early in the paper | 5 | A cross-sectional study was conducted. |
| Setting | 5 | Describe the setting, locations, and relevant dates, including periods of recruitment, exposure, follow-up, and data collection | 5 | Between August 2018 and August 2019, participants were recruited from Asian faith-based organizations and community-based organizations located in New York City. The detailed recruitment procedure is described elsewhere [18] and was approved by the institutional review board of NYU Grossman School of Medicine (IRB # i18-00268). |
| Participants | 6 | (*a*) *Cohort study*—Give the eligibility criteria, and the sources and methods of selection of participants. Describe methods of follow-up  *Case-control study*—Give the eligibility criteria, and the sources and methods of case ascertainment and control selection. Give the rationale for the choice of cases and controls  *Cross-sectional study*—Give the eligibility criteria, and the sources and methods of selection of participants | 4-5 | We recruited Asian American immigrant women currently living in New York City (NYC). We focused on the three largest Asian subgroups in NYC: East Asians (i.e., Chinese and Koreans), Southeast Asians (i.e., Filipinos), and South Asians (i.e., Bangladeshis) [21]. The eligibility criteria were (1) age between 18 and 75; (2) self-identified as an AAI woman born in one of four countries (i.e., China, Bangladesh, Philippines, South Korea) and immigrated to the U.S.; (3) not currently being treated with antihypertensive medications; (4) ability to read, speak, and write in English, Bangla, Chinese, or Korean; and (5) not currently pregnant. |
|  |  | (*b*) *Cohort study*—For matched studies, give matching criteria and number of exposed and unexposed  *Case-control study*—For matched studies, give matching criteria and the number of controls per case | n/a |  |
| Variables | 7 | Clearly define all outcomes, exposures, predictors, potential confounders, and effect modifiers. Give diagnostic criteria, if applicable | 5-7 | (page 5) In order to objectively measure the entire intensity spectrum of 24-hour activity, we used both wrist-accelerometry (a criterion measure of sleep in free-living settings) [23] and hip-accelerometry (a criterion measure of PA and sedentary behavior in free-living settings) [24]…. (page 7) This questionnaire has acceptable convergent validity with the chart-based Charlson Comorbidity Index (Spearman’s rho = 0.55) and excellent test-retest reliability (Intraclass Correlation Coefficient [ICC] = 0.94) [32]. |
| Data sources/ measurement | 8* | For each variable of interest, give sources of data and details of methods of assessment (measurement). Describe comparability of assessment methods if there is more than one group | *5-7* | Same as above |
| Bias | 9 | Describe any efforts to address potential sources of bias | 8 | We encountered 13% of missing self-report data (n = 11, 13%) which included demographic and clinical characteristics (age, education levels, marital status, and comorbidity index). The missing data were not missing at random; the majority of the missing data was from one Asian subgroup (Bangladeshi) due to their low levels of written Bengali literacy. Because the number of missing values for these variables would result in an unacceptable loss of cases in modeling, these variables were not included in modeling. In supplementary analyses, we re-ran all models while adjusting for these demographic variables in the reduced sample. |
| Study size | 10 | Explain how the study size was arrived at | 5 | We calculated the minimum sample size needed to detect the effect of replacing 30 minutes of sedentary time with MVPA on BMI. We used the effect estimate from a meta-analysis [22] which found that replacing 30 minutes of sedentary time with MVPA had a significant effect on BMI, with a pooled effect size of beta = –1.07 (95% CI = –1.80, –0.3). Based on this information, a minimum sample size of 80 was required to detect this effect within 95% CI with a power of > 0.8 to show the estimated required sample size for detecting these effects. |

| Quantitative variables | 11 | Explain how quantitative variables were handled in the analyses. If applicable, describe which groupings were chosen and why | 6-7 | (page 6) From these sleep periods, the minutes of “asleep” were summed to calculate sleep duration. Next, after excluding the sleep period from the hip accelerometer data, we applied the Choi et al. algorithm to identify non-wear time [26]. Freedson’s cutoff points were used to classify time spent in MVPA (≥1952 counts/min), light PA (101-1951 counts/min), and sedentary behavior (≤100 counts/min) [27] based on the valid wear time data from the hip accelerometer. For the analysis, participants who had a minimum of 4 days with 10 hours/day of wear time of both accelerometers from midnight-to-midnight were included in the current analyses…. (page 7) For analysis, all 24-hour activity variables were converted to 30 minutes/day units to aid in interpretation of the regression coefficients. All |
| --- | --- | --- | --- | --- |
| Statistical methods | 12 | (*a*) Describe all statistical methods, including those used to control for confounding | 7-8 | (page 7) All outcome variables were examined for normality and these variables approximated a normal distribution….(page 8) In supplementary analyses, we re-ran all models while adjusting for these demographic variables in the reduced sample. |
|  |  | (*b*) Describe any methods used to examine subgroups and interactions | 17 | Third, due to the small sample sizes, we were not able to test the potential differences in effects of behavior substitutions in Asian subgroups. |
|  |  | (*c*) Explain how missing data were addressed | 8 | We encountered 13% of missing self-report data (n = 11, 13%) which included demographic and clinical characteristics (age, education levels, marital status, and comorbidity index). The missing data were not missing at random; the majority of the missing data was from one Asian subgroup (Bangladeshi) due to their low levels of written Bengali literacy. Because the number of missing values for these variables would result in an unacceptable loss of cases in modeling, these variables were not included in modeling. In supplementary analyses, we re-ran all models while adjusting for these demographic variables in the reduced sample. |
|  |  | (*d*) *Cohort study*—If applicable, explain how loss to follow-up was addressed  *Case-control study*—If applicable, explain how matching of cases and controls was addressed  *Cross-sectional study*—If applicable, describe analytical methods taking account of sampling strategy | n/a |  |
|  |  | (*e*) Describe any sensitivity analyses | 8 | Same as 12 (*c*) |
| Results | | | | |
| Participants | 13* | (a) Report numbers of individuals at each stage of study—eg numbers potentially eligible, examined for eligibility, confirmed eligible, included in the study, completing follow-up, and analysed | 8 | Eighty-six participants were eligible for the study of which 75 (87%) completed both hip and wrist accelerometer monitoring for at least 4 days and were included in the analysis. |
|  |  | (b) Give reasons for non-participation at each stage | n/a | We could not provide specific numbers for those potentially eligible or examined for eligibility, as our participant recruitment was conducted through community-based events and organizations. |
|  |  | (c) Consider use of a flow diagram | n/a |  |
| Descriptive data | 14* | (a) Give characteristics of study participants (eg demographic, clinical, social) and information on exposures and potential confounders | 9-10 | Table 1 |
|  |  | (b) Indicate number of participants with missing data for each variable of interest | 8 | We encountered 13% of missing self-report data (n = 11, 13%) which included demographic and clinical characteristics (age, education levels, marital status, and comorbidity index). |
|  |  | (c) *Cohort study*—Summarise follow-up time (eg, average and total amount) | n/a |  |
| Outcome data | 15* | *Cohort study*—Report numbers of outcome events or summary measures over time | n/a |  |
|  |  | *Case-control study—*Report numbers in each exposure category, or summary measures of exposure | n/a |  |
|  |  | *Cross-sectional study—*Report numbers of outcome events or summary measures |  | Table 1-4 |
| Main results | 16 | (*a*) Give unadjusted estimates and, if applicable, confounder-adjusted estimates and their precision (eg, 95% confidence interval). Make clear which confounders were adjusted for and why they were included | 10-13 | Table 2-4 |
|  |  | (*b*) Report category boundaries when continuous variables were categorized | n/a |  |
|  |  | (*c*) If relevant, consider translating estimates of relative risk into absolute risk for a meaningful time period | n/a |  |

| Other analyses | 17 | Report other analyses done—eg analyses of subgroups and interactions, and sensitivity analyses | 14 | The adjusted model indicated that results remained consistent when we adjusted the BMI, waist circumference, and blood pressure models for age, education level, marital status, and comorbidity. The detailed results are included in S1 Table. |
| --- | --- | --- | --- | --- |
| Discussion | | | | |
| Key results | 18 | Summarise key results with reference to study objectives | 14 | To our knowledge, this is the first study showing the effects of reallocating 24-hour activity behaviors on CVD risk factors in diverse subgroups of AAI women. In this study, isotemporal substitution models demonstrated a clinically relevant effect of replacing sedentary behavior or light PA with either sleep or MVPA in AAI women. Considering that our sample of AAI women spent an average of 30 minutes in MVPA per day but only spent 5.3 hours in sleep, replacing 30 minutes of sedentary time with sleep might be a more feasible strategy to improve cardiovascular risk than encouraging them to engage in more MVPA. |
| Limitations | 19 | Discuss limitations of the study, taking into account sources of potential bias or imprecision. Discuss both direction and magnitude of any potential bias | 16-17 | (page 16) This study has some limitations and interpretation of the findings must be made with caution….(page 17) Lastly, although we proportionally recruited the three largest Asian subgroups in NYC, our findings from our studies have a limited generalizability given that women who participate in the community-based organizations we recruited from may have different physical activity routines than those who do not participate in such organizations. |
| Interpretation | 20 | Give a cautious overall interpretation of results considering objectives, limitations, multiplicity of analyses, results from similar studies, and other relevant evidence | 14-16 | See discussion |
| Generalisability | 21 | Discuss the generalisability (external validity) of the study results | 16-17 | Second, our findings should be interpretated with caution due to substantial missing self-administered survey data from the Bangladeshi subgroup. The issue of incomplete Bengali surveys was addressed by omitting significantly impacted demographic characteristics from isotemporal substitution models and conducting sensitivity analyses afterward. We also compared survey-returner vs. non-returner characteristics and found no significant difference at baseline in each of the 24-hour activity components. Third, due to the small sample sizes, we were not able to test the potential differences in effects of behavior substitutions in Asian subgroups. Lastly, although we proportionally recruited the three largest Asian subgroups in NYC, our findings from our studies have a limited generalizability given that women who participate in the community-based organizations we recruited from may have different physical activity routines than those who do not participate in such organizations. |
| Other information | |  | | |
| Funding | 22 | Give the source of funding and the role of the funders for the present study and, if applicable, for the original study on which the present article is based | In funding page | CP received the American Heart Association’s Go Red For Women Collaborative Grant 16SFRN27810006 (https://professional.heart.org/en/). The funders had no role in study design, data collection and analysis, decision to publish, or preparation of the manuscript.  SK received the National Institutes of Health, National Institute for Minority Health and Health Disparities-funded Center for the Study of Asian American Health U54 MD000538. (https://www.nimhd.nih.gov/). The funders had no role in study design, data collection and analysis, decision to publish, or preparation of the manuscript. |

*Give information separately for cases and controls in case-control studies and, if applicable, for exposed and unexposed groups in cohort and cross-sectional studies.

**Note:** An Explanation and Elaboration article discusses each checklist item and gives methodological background and published examples of transparent reporting. The STROBE checklist is best used in conjunction with this article (freely available on the Web sites of PLoS Medicine at http://www.plosmedicine.org/, Annals of Internal Medicine at http://www.annals.org/, and Epidemiology at http://www.epidem.com/). Information on the STROBE Initiative is available at www.strobe-statement.org.
