# Supplementary material for: Reallocating time between device-measured 24-hour activities and cardiovascular risk in Asian American immigrant women: An isotemporal substitution model
Source: PLoS One. 2024 Jan 10;19(1):e0297042. doi: 10.1371/journal.pone.0297042 (PMC10781047; doi:10.1371/journal.pone.0297042)
Supplement: S1 Table — (DOCX) [file pone.0297042.s002.docx]

|  |  |  |  |  |
| --- | --- | --- | --- | --- |

**S1 Table. Supplementary analyses of isotemporal substitution models for cardiovascular risk factors, adjusting for demographic and clinical characteristics (n=63)**

| Model | Sedentary | Light PA | MVPA | Sleep |
| --- | --- | --- | --- | --- |
| BMI | | | | |
| Replace sedentary | NA | 0.26 (-0.05, 0.57) | -1.51 (-2.71, -0.31)* | -0.65 (-1.18, -0.11)* |
| Replace light PA | -0.27 (-0.58, 0.04) | NA | -1.78 (-3.1, -0.46)** | -0.91 (-1.46, -0.37)** |
| Replace MVPA | 1.47 (0.27, 2.67)* | 1.73 (0.41, 3.05)* | NA | 0.82 (-0.55, 2.2) |
| Replace sleep | 0.64 (0.1, 1.17)* | 0.9 (0.35, 1.45)** | -0.88 (-2.25, 0.5) | NA |
| Waist circumference | | | | |
| Replace sedentary | NA | 0.56 (-0.32, 1.43) | -4.62 (-8.02, -1.22)** | -1.62 (-3.15, -0.09)* |
| Replace light PA | -0.57 (-1.45, 0.31) | NA | -5.2 (-8.94, -1.46)** | -2.19 (-3.74, -0.64)** |
| Replace MVPA | 4.45 (1.04, 7.86)* | 4.99 (1.24, 8.74)* | NA | 2.83 (-1.08, 6.74) |
| Replace sleep | 1.59 (0.06, 3.12)* | 2.15 (0.6, 3.7)** | -3.03 (-6.93, 0.86) | NA |
| Systolic blood pressure | | | | |
| Replace sedentary | NA | -0.52 (-1.93, 0.9) | -1.46 (-6.93, 4.02) | -0.68 (-3.15, 1.78) |
| Replace light PA | -0.52 (-1.93, 0.9) | NA | -1.99 (-8.01, 4.04) | -1.2 (-3.69, 1.3) |
| Replace MVPA | 1.33 (-4.13, 6.79) | 1.82 (-4.18, 7.83) | NA | 0.64 (-5.62, 6.91) |
| Replace sleep | 0.66 (-1.8, 3.11) | 1.17 (-1.32, 3.66) | -0.81 (-7.07, 5.46) | NA |
| Diastolic blood pressure | |  |  |  |
| Replace sedentary | NA | 0.55 (-0.46, 1.56) | -1.1 (-5, 2.79) | -0.26 (-2.02, 1.49) |
| Replace light PA | -0.55 (-1.56, 0.46) | NA | -1.65 (-5.94, 2.63) | -0.81 (-2.59, 0.96) |
| Replace MVPA | 1.11 (-2.77, 4.99) | 1.66 (-2.61, 5.93) | NA | 0.85 (-3.61, 5.31) |
| Replace sleep | 0.27 (-1.48, 2.01) | 0.81 (-0.96, 2.58) | -0.84 (-5.29, 3.62) | NA |

*Note.* Values represent unstandardized regression coefficients (95% Confidence Intervals). Covariates = age, comorbidity index, education level (college or higher), and marital/partner status (yes/no).

Abbreviation: NA=not applicable, PA=physical activity, MVPA=Moderate-to-vigorous physical activity

**p* < 0.05, ***p* < 0.01.
